# Clinical Manifestations, Antifungal Drug Susceptibility, and Treatment Outcomes for Emerging Zoonotic Cutaneous Sporotrichosis, Thailand

**DOI:** 10.3201/eid3012.240467

**Published:** 2024-12

**Authors:** Pattriya Jirawattanadon, Sumanas Bunyaratavej, Charussri Leeyaphan, Piriyaporn Chongtrakool, Panitta Sitthinamsuwan, Waratchaya Panjapakkul, Suthasanee Prasertsook, Phuwakorn Saengthong-aram, Nicha Wareesawetsuwan, Julaluck Posri, Penvadee Pattanaprichakul

**Affiliations:** Siriraj Hospital Faculty of Medicine, Bangkok, Thailand

**Keywords:** sporotrichosis, *Sporothrix*, subcutaneous fungal infection, drug susceptibility, itraconazole, fungi, respiratory infections, zoonoses, Thailand

## Abstract

We analyzed clinical manifestations, antifungal susceptibility, and treatment outcomes of cutaneous sporotrichosis in Thailand during 2018–2022. The study included 49 patients whose mean age was 58.7 (SD 16.9) years; 65.3% were female and 34.7% male. A history of cat exposure was reported in 32 (65.3%) patients who had a significantly higher prevalence of upper extremity lesions than did those without cat contact (90.6% vs. 41.7%; adjusted odds ratio 18.9 [95% CI 3.2–92.9]). Among patients >60 years of age, lesions were more likely to be nonpustular than for patients <60 years of age (82.1% vs. 52.4%; p = 0.033). All 9 isolates tested for antifungal drug susceptibility exhibited an itraconazole MIC of <1 μg/mL. Oral itraconazole monotherapy was effective; the median time-to-cure was 180 days (interquartile range 141–240 days). Physicians should heighten their awareness of potential sporotrichosis causes, particularly when a history of animal contact exists.

Sporotrichosis is a chronic infection caused by *Sporothrix* spp., dimorphic fungi that exist in a hyphal form at low temperatures or as budding yeast at high temperatures. This pathogen is found in soil, plants, and organic matter and gives rise to an anthropozoonotic disease primarily affecting the skin and subcutaneous tissues after traumatic inoculation by contaminated materials (*1*–*3*). *Sporothrix* spp. have been previously classified under the *S. schenckii* complex, which comprise *S. schenckii* sensu stricto, *S. brasiliensis*, *S. globosa*, *S. luriei*, *S. pallida*, *S. mexicana*, and *S. chilensis*. The classification system has been updated to include a clinical clade consisting of *S. schenckii*, *S. globosa*, *S. brasiliensis*, and *S. luriei*. Species from the environmental clade, including *S. pallida*, *S. mexicana*, and *S. chilensis*, rarely cause human infection (*4*).

Although sporotrichosis has a global distribution, clinical and epidemiologic features vary. It is endemic in regions that have tropical and subtropical climates and maintain high humidity and 25°C–28°C temperatures (*2*,*5*,*6*). Several reports have cited case series or outbreak instances among occupational groups prone to minor soil- and plant-inflicted injuries, such as florists, miners, foresters, and gardeners (*7*–*12*). Furthermore, since the 1980s, owning cats has emerged as a risk factor for zoonotic transmission of sporotrichosis (*13*,*14*). Human sporotrichosis outbreaks have been linked to scratches or bites from infected cats. Domestic cats, because of their pathogen-rich lesions and intimate interactions with humans, serve as *Sporothrix* spp. vectors (*15*–*20*). 

The disease has an incubation period ranging from a few days to several months (*21*) and can manifest in cutaneous, pulmonary, and disseminated forms. Cutaneous manifestation is the most common, typically occurring as erythematous papules or pustules that form ulcerated nodules involving local lymphatic channels, which cause lymphangitis and lymphadenopathy (*2*,*22*). Sporotrichosis can also be categorized into fixed cutaneous, lymphocutaneous, and disseminated cutaneous forms. Although the fixed and lymphocutaneous forms are commonly observed, disseminated cutaneous sporotrichosis is rarely reported except in immunosuppressed patients (*23*). Fungi isolation is the standard for diagnosis; the first-line treatment is itraconazole (*24*). For antifungal susceptibility testing of *Sporothrix* spp., broth microdilution, as recommended by the Clinical and Laboratory Standards Institute (https://clsi.org/standards/products/microbiology/documents/m38), is the primary protocol used to determine in vitro MICs for drugs used in sporotrichosis treatment. Itraconazole is the most effective drug for treating sporotrichosis. A cutoff point of 1 μg/mL is used according to a previous study correlating the itraconazole MIC with clinical outcomes (*25*). Terbinafine has demonstrated the lowest geometric mean MIC, followed by ketoconazole (*26*). No associations have been found between higher amphotericin B or itraconazole MIC values and unfavorable outcomes (*25*).

Sporotrichosis is a relatively rare disease in Thailand. However, since 2018, a substantial increase in the number of sporotrichosis patients has been observed at the outpatient dermatology clinic at Siriraj Hospital in Bangkok, Thailand. This surge parallels the growing evidence of feline sporotrichosis observed in Thailand since 2018 (*27*). We investigated the clinical manifestations and treatment outcomes for a recent emerging outbreak of cutaneous sporotrichosis in Thailand. 

## Methods

We conducted a retrospective study of patients who had a cutaneous sporotrichosis diagnosis at the dermatology clinic of Siriraj Hospital during January 1, 2018–August 27, 2022. The Siriraj Hospital Institutional Review Board authorized the study protocol (approval nos. Si 979/2020 and Si 671/2021). We enrolled patients in the study if the diagnosis was confirmed by positive isolation of *S. schenckii* complex in culture. We extracted patient sex, age, duration of symptoms, underlying medical conditions, occupation, exposure history (animal contact, injury history, and contamination with soil), clinical manifestations, histopathology, laboratory culture, susceptibility testing of the causative fungus, treatment options, and treatment outcomes from medical records. We considered patients to be immunocompromised if they exhibited specific conditions that can cause host defense defects, such as cirrhosis, diabetes mellitus, or recent chemotherapy (*28*).

Hospital staff collected specimens for histopathologic examination and laboratory culture; specimens were collected from tissue biopsies or pus from cutaneous lesions. Fungi were identified by phenotypic characteristics, including colony morphology, conidial arrangement, and physiologic traits (carbohydrate assimilation of dextrose, sucrose, and raffinose), as well as temperature tolerance at 37°C and 40°C. Antifungal susceptibility testing was undertaken in some cases on the basis of convenience selection by using the Clinical and Laboratory Standards Institute broth dilution method, as noted . Testing was performed for 5-flucytosine, amphotericin B, anidulafungin, caspofungin, micafungin, fluconazole, itraconazole, posaconazole, and voriconazole.

We defined an improved lesion as a lesion that had become smaller or drier without cutaneous extension and a cure as a complete healing of lesions with or without scarring or hyperpigmentation. We conducted telephone interviews to assess patients who had incomplete data because of loss to follow-up during the treatment course. 

### Statistical Analysis

We used descriptive statistics for demographic data, clinical manifestations, histopathologic examinations, laboratory findings, antifungal susceptibility, treatments, and treatment outcomes. We calculated means and SDs for normally distributed continuous data, medians and interquartile ranges (IQRs) for non–normally distributed continuous data, and numbers and percentages for categorical data. We used χ^2^ and Fisher exact tests to compare clinical characteristics between patients who had or did not have exposure to cats. We used a binary logistic regression model and forward stepwise selection method to estimate the odds ratio (OR) of a binary response. We constructed a Cox proportional hazards model to determine the hazard ratio for each factor and to measure time-to-improve and time-to-cure. We adjusted for patient sex and age in the multivariable analysis of both binary logistic regression and Cox proportional hazards models. In addition, we calculated the OR, hazard ratio, and 95% CI for each pertinent variable. We analyzed data by using PASW Statistics 18.0 (SPSS, https://www.sbas.com.hk). A p value of <0.05 indicated statistical significance.

## Results

**Figure 1:**
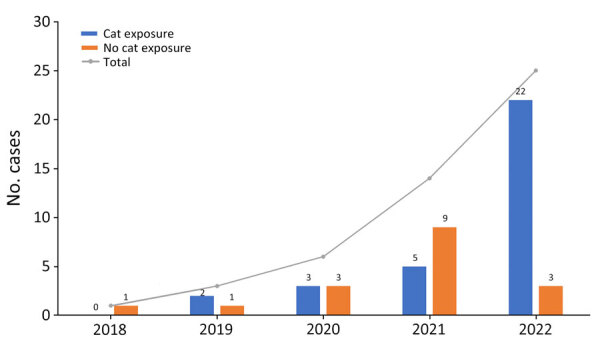
Numbers of sporotrichosis cases during 2018–2022 in study of clinical manifestations, antifungal drug susceptibility, and treatment outcomes for emerging zoonotic cutaneous sporotrichosis, Thailand. Line indicates the total number of cases each year. Numbers above each bar indicate the number of patients exposed or not exposed to cats.

We included 49 patients who had cutaneous sporotrichosis during 2018–2022 and summarized their clinical characteristics (Table 1). We observed a marked increase in cases each year during this period, escalating from 1 case to 3 cases, then to 6 cases, 14 cases, and finally 25 cases (Figure 1). The mean age of patients was 58.7 (SD 16.9) years; 32 (65.3%) were female and 17 (34.7%) male. Only 9 patients were immunocompromised, having diabetes mellitus or sigmoid colon cancer with recent chemotherapy. The median duration from lesion onset to diagnosis was 30 (range 7–180) days. Before sporotrichosis diagnosis, 15 (30.6%) patients had undergone treatment regimens for nontuberculous mycobacterial infections but had no observed improvement.

**Table 1:** Patient demographics in study of clinical manifestations, antifungal drug susceptibility, and treatment outcomes for emerging zoonotic cutaneous sporotrichosis, Thailand*

Demographic data	Sporotrichosis cases, n = 49
Patient sex	
F	32 (65.3)
M	17 (34.7)
Mean age, y (±SD)	58.7 (±16.9)
Median symptom duration, d (IQR)	30 (21–60)
Immune status	
Immunocompetent host	40 (81.6)
Immunocompromised host	9 (18.4)
Diabetes mellitus	8/9 (88.9)
Sigmoid carcinoma and recent chemotherapy	1/9 (11.1)
Occupation	
Retired/unemployed	15 (30.6)
Officer	10 (20.4)
Merchant/business owner	8 (16.3)
Veterinarian/veterinary assistants	4 (8.2)
Student	4 (8.2)
Housekeeper/cleaner	4 (8.2)
Other†	4 (8.2)
Exposure history	
Zoonosis	35 (71.4)
Cats	32/35 (91.4)
Insect bite	3/35 (8.6)
Wound contact with soil	1 (2.0)
Unknown	13 (26.5)
Family history of cutaneous sporotrichosis	5 (10.2)
Clinical symptoms	
Painless	24 (49.0)
Painful	19 (38.8)
Itching	6 (12.2)
Lesions	
Median no. lesions (IQR)	2.0 (1.0–3.0)
Single	20 (40.8)
Multiple	29 (59.2)
Unilateral	25/29 (86.2)
Bilateral	4/29 (13.8)
Morphology	
Nonpurulent, nonulcerative papule, plaque, or nodule	34 (69.4)
Purulent, ulcerative pustule, ulcer, abscess	15 (30.6)
Arrangement	
Satellite nodules around the ulcer rim	27 (55.1)
Lymphocutaneous pattern	22 (44.9)
Lesion site	
Head and neck	3 (6.1)
Trunk	1 (2.0)
Upper extremities	36 (73.5)
Lower extremities	11 (22.4)
Lymphadenopathy, n = 44	6/44 (13.6)
Histopathology, n = 44	
Mixed cell, suppurative granuloma	36/44 (81.8)
Nonspecific granulomatous inflammation	3/44 (6.8)
Evidence of fungus observed	0/44 (0.0)
Outcome	
Completely cured	41 (83.7)
Lost to follow-up	8 (16.3)
Treatment duration	
Median duration until improved, d (IQR)	46 (30–90)
Mean duration until cured, d (IQR), n = 41	180 (141–240)

*Values are no. patients/total no. patients (%) except as indicated. IQR, interquartile range. †Other occupations included teacher, monk, and boat driver.

Thirty-five (71.4%) patients reported a history of animal exposure; 32 had been exposed to cats and 3 to insects. Of the 3 patients with insect bites, 2 had bites from mosquitoes and 1 had an unidentified insect bite. For all 3 patients, lesions developed later on the lower extremities at the sites of those insect bites. Among the patients exposed to cats, 23 had direct contact, and 22 (95.7%) of those 23 reported scratches or bites (Table 2). Most (27/32) patients exposed to cats had contact with their own cats, and all let their cats roam outdoors. In addition, 21 (65%) patients had been in direct contact with cats that had sporotrichosis diagnosed by a veterinarian. Although 15 (30.6%) patients were retired or unemployed, 4 (8.2%) patients were employed in veterinary professions; 2 were veterinarians and 2 veterinary assistants. All 4 of those patients were scratched or bitten by cats that had sporotrichosis. Only 2/49 (4.1%) patients had a history of gardening, and none reported exposure to rose thorns. One patient reported falling from a bicycle onto soil causing an abrasion wound on his left leg, which later developed into lymphocutaneous lesions. Sporotrichosis developed in 13/49 (26.5%) patients who had no recognized risk factors.

**Table 2:** Patient exposure to cats in study of clinical manifestations, antifungal drug susceptibility, and treatment outcomes for emerging zoonotic cutaneous sporotrichosis, Thailand*

Patient exposures to cats	No. sporotrichosis cases/total (%)
Direct contact	23/32 (71.9)
Being scratched or bitten	22/23 (95.7)
Feeding	16/23 (69.6)
Attending to cat wound	4/23 (17.4)
Indirect contact†	9/32 (28.1)
Types of cat socialization	
Owned, outdoor cat‡	27/32 (84.4)
Stray cat	5/32 (15.6)
Exposed to cat with sporotrichosis diagnosis	21/32 (65.6)

*Patients could have >1 contact history. †Patients reported stray cats in the vicinity of their homes but indicated no direct contact. ‡Cats that have homes but can go outside.

**Figure 2:**
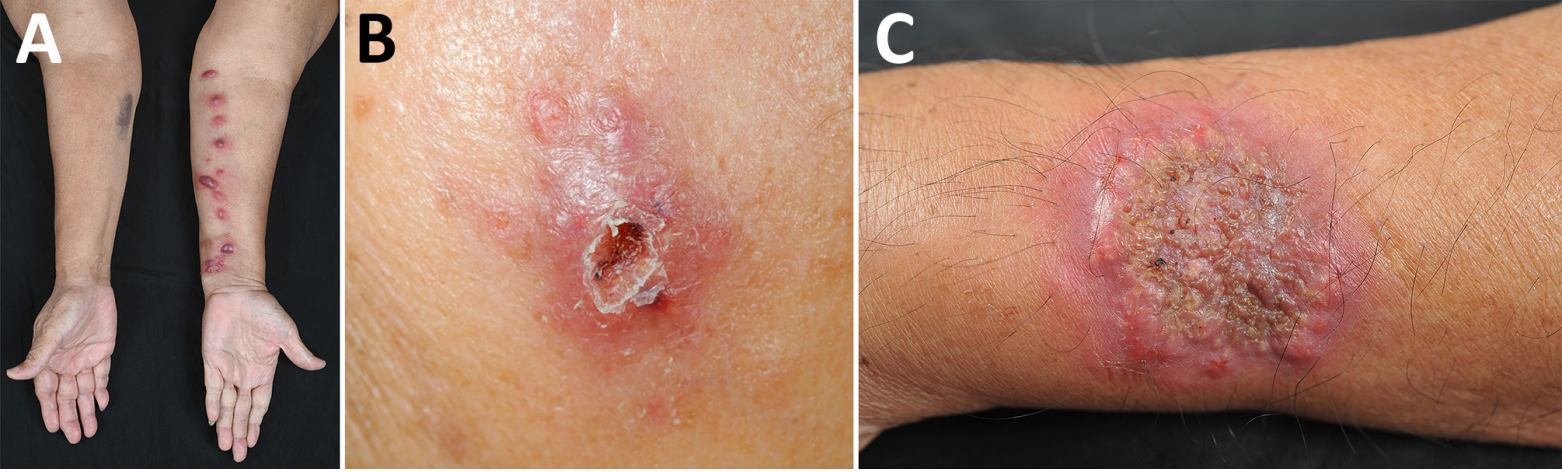
Clinical disease manifestations in study of antifungal drug susceptibility and treatment outcomes for emerging zoonotic cutaneous sporotrichosis, Thailand. A) Lymphocutaneous pattern on a patient’s left forearm; B) satellite nodules encircling ulcer rim on a patient’s left cheek; C) verrucous plaque on a patient’s left forearm.

No pain was experienced in 49.0% of patients. Multiple lesions were observed in 59.2% of cases manifesting a lymphocutaneous pattern (44.9%; Figure 2, panel A). Fifteen (30.6%) patients had purulent discharge and ulceration. Single lesions, which mostly had satellite nodules around the ulcer rim, were found in 40.8% of patients (Figure 2, panel B). Three (6.1%) patients had solitary or multiple verrucous plaques (Figure 2, panel C). Most (73.5%) lesions were located on the upper extremities, and only 13.6% of patients manifested lymphadenopathy. Patients >60 years of age also had significantly more nonpustular lesions than younger patients (23/28 [82.1%] vs. 11/21 [52.4%]; p = 0.033).

Patients with a history of cat contact exhibited higher levels of immunocompetence (90.6% vs. 64.7%; adjusted OR 5.2 [95% CI 1.1–24.8]), and lesions were more frequently located on the upper extremities (90.6% vs. 41.7%; adjusted OR 18.9 [95% CI 3.2–92.9]) than in those who had no cat exposure (Table 3). The difference in lesion frequency was also statistically significant in multivariate analysis. In addition, lesions tended to be multiple and arranged in a lymphocutaneous pattern in patients who had cat contact.

**Table 3:** Clinical manifestation comparisons between patients with and without cat exposure in study of clinical manifestations, antifungal drug susceptibility, and treatment outcomes for emerging zoonotic cutaneous sporotrichosis, Thailand*

Demographic data	Cat exposure, n = 32	No cat exposure, n = 17	Univariate analysis			Multivariate analysis†	
			Crude OR (95% CI)	p value		Adjusted OR‡ (95% CI)	p value
Patient sex							
M	9 (28.1)	8 (47.1)	2.3 (0.7–7.7)	0.189		NA	
F	23 (71.9)	9 (52.9)	Referent				
Mean age, y (±SD)	55.0 (±16.4)	65.5 (±16.1)	0.9 (0.9–1.0)	0.084		1.0 (0.9–1.0)	0.095
Median symptom duration, d (IQR)	30.0 (21.0–83.0)	30.0 (22.0–60.0)	1.0 (0.9–1.0)	0.916		NA	
Immunologic status							
Immunocompromised	3 (9.4)	6 (35.3)	5.3 (1.1–24.8)	**0.036**		5.2 (1.1–24.8)	**0.036**
Immunocompetent	29 (90.6)	11 (64.7)	Referent			Referent	
Sporotrichosis in family member, n = 47	5/32 (15.6)	0/15 (0.0)	NA	0.162		NA	
Lesions							
Single	9 (28.1)	11 (64.7)	Referent	**0.016**		Referent	0.100
Multiple	23 (71.9)	6 (35.3)	4.7 (1.3–16.5)			6.1 (0.9–24.4)	
Lesions site							
Head and neck	0 (0)	3 (17.6)	NA	0.073		NA	
Trunk	0 (0.0)	1 (5.9)	NA	0.347		NA	
Upper extremities	29 (90.6)	7 (41.2)	13.8 (3.0–63.9)	**0.001**		18.9 (3.2–92.9)	**0.001**
Lower extremities	5 (15.6)	6 (35.3)	0.3 (0.1–1.4)	0.125		NA	
Morphology							
Nonpustular	23 (71.9)	11 (64.7)	Referent	0.605		NA	
Pustular	9 (28.1)	6 (35.3)	0.7 (0.2–2.5)				
Arrangement							
Lymphocutaneous pattern	18 (56.3)	4 (23.5)	4.2 (1.1–15.7)	**0.034**		3.7 (0.8–16.7)	0.093
Lymphadenopathy, n = 44	3/28 (10.7)	3/16 (18.8)	0.5 (0.1–3.0)	0.460			
Median treatment duration, d (IQR)							
Until improvement	46.0 (30.0–107.0)	53.0 (30.0–90.0)	1.0 (0.9–1.0)	0.625		NA	
Until cured	183.0 (135.0–246.0)	165.0 (136.0–206.0)	1.0 (0.9–1.0)	0.495		NA	

*Values are no. patients/total no. patients (%) except as indicated. Bolded numbers are significant p values <0.05. IQR, interquartile range; NA, not applicable; OR, odds ratio. Multivariate analysis was conducted by using multiple binary logistic regression. ‡Adjusted for patient sex and age.

Histopathologic analysis was conducted for 44 of 49 patients and revealed mixed cell or suppurative granuloma in 81.8% of lesions and nonspecific granulomatous inflammation in 6.8% of lesions. Periodic acid Schiff and Gomori methenamine silver stains were performed in all cases. No evidence of fungi was detected. Only 1 patient did not undergo a skin biopsy; their sporotrichosis diagnosis was confirmed through a positive pus culture result. Antifungal susceptibility testing for 9 patients was reported, and all 9 isolates exhibited an itraconazole MIC of <1 μg/mL (Table 4).

**Table 4:** Antifungal susceptibility test results for 9 patients in study of clinical manifestations, antifungal drug susceptibility, and treatment outcomes for emerging zoonotic cutaneous sporotrichosis, Thailand*

Antifungal drug	MIC, μg/mL								
	Patient 1	Patient 2	Patient 3	Patient 4	Patient 5	Patient 6	Patient 7	Patient 8	Patient 9
5-Flucytosine	0.5	32	1	32	2	2	2	2	2
Amphotericin B	2	2	4	1	4	4	4	2	2
Anidulafungin	4	>8	>8	1	>8	0.5	1	>8	0.5
Caspofungin	>8	>8	>8	>8	>8	4	>8	>8	1
Fluconazole	>256	256	32	256	256	256	>256	256	>256
Itraconazole	1	0.25	0.5	0.5	0.25	1	1	0.5	0.25
Micafungin	>8	>8	>8	>8	>8	>8	>8	>8	>8
Posaconazole	0.5	0.5	0.5	1	0.25	0.25	0.25	0.25	0.25
Voriconazole	0.12	0.25	0.06	1	0.03	0.06	0.5	0.25	0.06

*Sporothrix schenckii complex fungi isolated from patient specimens were tested for susceptibility to antifungal drugs.

Of the 49 patients, 41 received itraconazole and 5 terbinafine as first-line treatment. The remaining 3 patients were lost to follow-up before beginning treatment. Treatment outcomes could only be observed in 41 patients; the remaining 8 were lost to follow-up. All itraconazole-treated patients received an initial dose of 200 mg/day. After ≈2 months of treatment, the itraconazole dose was increased to 400 mg/day in 11 (26.8%) patients because of worsening lesions. All 11 patients entered remission within a median of 127 (IQR 60–203) days after the dose increase. Antifungal susceptibility testing had been performed for 2 of those 11 patients (patients 2 and 7; Table 4), 1 of whom failed to improve after 4 months of increased itraconazole dose and was, therefore, treated by excision, followed by another 7 weeks of itraconazole and remission. We did not observe a significant difference in itraconazole MICs between patients who had treatment failure and those who had successful treatment at the dose of 200 mg/day (median MIC 1.0 [IQR 0.25–1.0] μg/mL vs. 0.5 [IQR 0.25–0.625] μg/mL; p = 0.414). No patients reported serious side effects from itraconazole.

**Figure 3:**
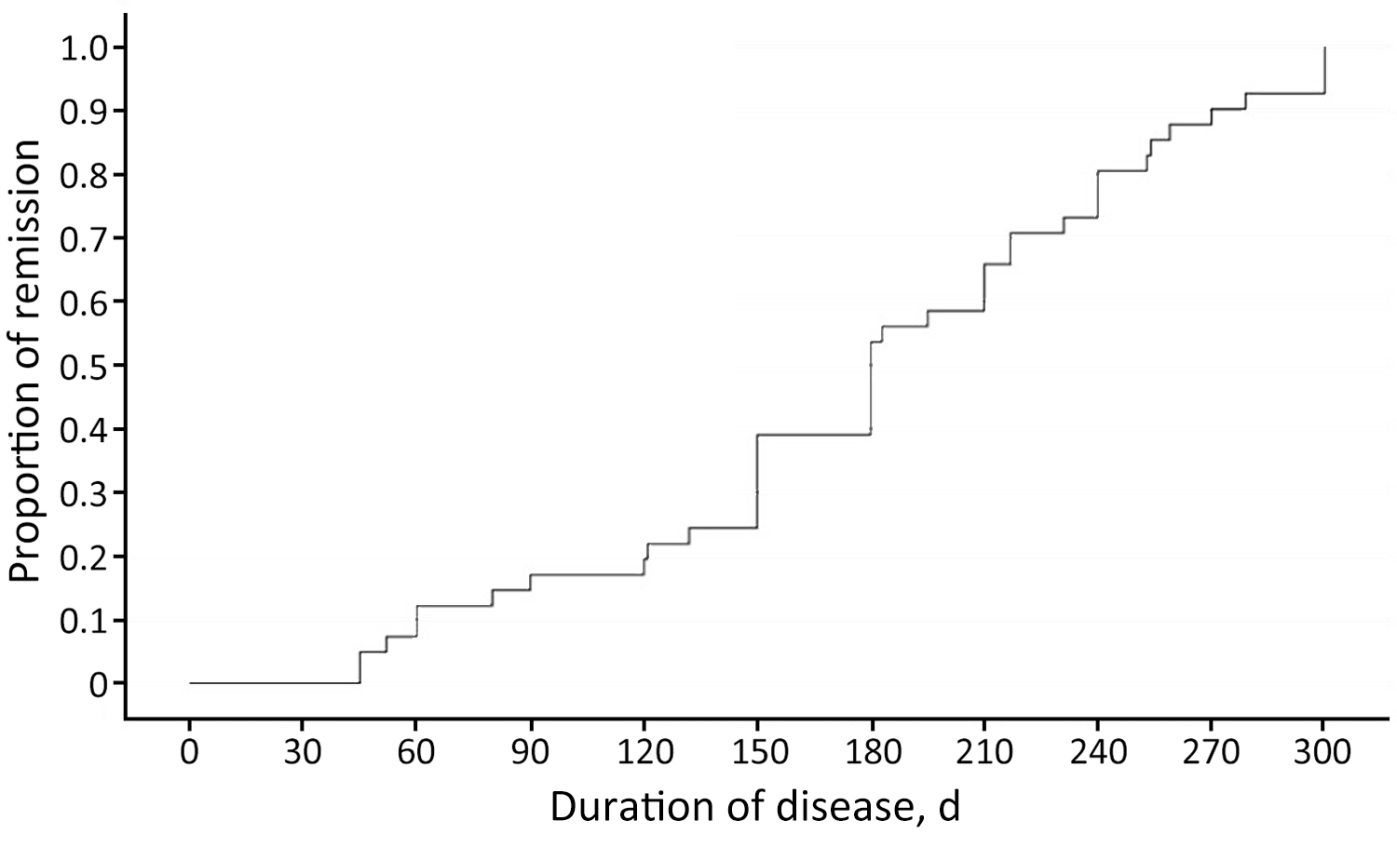
Proportion of patients in remission over time in study of clinical manifestations, antifungal drug susceptibility, and treatment outcomes for emerging zoonotic cutaneous sporotrichosis, Thailand. Kaplan–Meier survival analysis was used to determine the duration of cutaneous sporotrichosis in patients.

The median time-to-improvement was ≈46 (IQR 30–90) days, and the median time-to-cure was 180 (IQR 141–240) days. According to the Cox proportional hazards model, no variable was significantly associated with improvement or cure rates (Table 5). By 6 months after treatment, remission had been achieved in 50% of patients, and by 8 months, ≈75% of patients had entered remission (Figure 3).

**Table 5:** Comparison of hazard ratios for time-to-improvement and time-to-cure among patients in study of clinical manifestations, antifungal drug susceptibility, and treatment outcomes for emerging zoonotic cutaneous sporotrichosis, Thailand*

Variables	Time-to-improvement†			Time-to-cure				
	Univariate analysis			Univariate analysis			Multivariate analysis	
	Crude HR (95% CI)	p value		Crude HR (95% CI)	p value		Adjusted HR‡ (95% CI)	p value
Patient sex								
M	Referent	0.452		Referent	0.102		Referent	0.620
F	1.3 (0.7–2.5)			1.8 (0.9–3.7)			1.9 (0.9–4.1)	
Patient age at diagnosis, y								
<60	Referent	0.587		Referent	0.176		Referent	0.109
>60	1.2 (0.7–2.1)			1.5 (0.8–2.9)			1.7 (0.9–3.1)	
Immunologic status								
Immunocompromised	1.5 (0.7–3.4)	0.299		0.8 (0.4–2.0)	0.686		NA	
Immunocompetent	Referent			Referent			NA	
Cat exposure								
Yes	0.9 (0.5–1.7)	0.648		0.7 (0.4–1.4)	0.349		NA	
No	Referent			Referent			NA	
Lesions								
Single	Referent	0.253		Referent	0.761		NA	
Multiple	0.7 (0.4–1.3)			1.1 (0.6–2.1)			NA	
Morphology								
Nonpustular	Referent	0.445		Referent	0.448		NA	
Pustular	0.8 (0.4–1.5)			1.3 (0.7–2.5)			NA	
Arrangement								
Lymphocutaneous pattern	0.9 (0.5–1.8)	0.773		1.2 (0.6–2.3)	0.559		NA	
Lymphadenopathy	0.9 (0.6–2.4)	0.895		2.6 (0.9–7.1)	0.086		2.7 (1.0–7.5)	0.106
Itraconazole susceptibility§								
MIC <1 μg/mL	Referent	0.735		Referent	0.998		NA	
MIC = 1 μg/mL	1.3 (0.3–5.9)			1.1 (0.2–4.3)			NA	

*HRs were calculated by using the Cox proportional hazard model. HR, hazard ratio; NA, not applicable. †Multivariate analysis was not performed for time-to-improvement variables. ‡Adjusted for patient age and sex. §Itraconazole susceptibility tests were performed for specimens from 9 patients. A cutoff point of 1 μg/mL was used according to a previous study relating the itraconazole MIC to clinical outcomes (*26*).

## Discussion

Four human cases of sporotrichosis have previously been reported in Thailand (*29*–*31*). This study documents a high number of patients with sporotrichosis reported in the country during 2018–2022. The historically low reported incidence might have been from underreporting or underdiagnosis, possibly because of limited laboratory capabilities in Thailand (*29*). 

Sporotrichosis is endemic in various regions worldwide (*32*), including Latin America, South Africa, Australia, and several countries in Asia, such as India, China, Japan, and Malaysia (*33*). The largest known zoonotic outbreak since the 20th Century was reported in Brazil (*32*). In Thailand, sporotrichosis cases have rarely been documented before 2018; our hospital typically encountered an average of only 1 case per year, initially linked to a case series reported in another hospital (*29*). However, since 2018, sporotrichosis has been increasingly reported in stray cats in Thailand (*34*), accompanied by a corresponding rise in human disease diagnosed at our hospital. Laboratory detection methods have remained consistent; conventional fungal detection techniques have been used routinely at our facility because of their cost-effectiveness for clinical services, whereas molecular techniques are primarily reserved for research purposes. Therefore, the increase in case numbers is more likely attributable to a true outbreak. 

More women were enrolled in this study, ≈2 times more women than men. This ratio aligns with most other sporotrichosis outbreak reports, which have indicated similar percentages of women in study populations, ranging from 53% to 72% (*35*–*38*). The most commonly reported mean or median age has been 40–50 years (*35*–*38*), which is younger than that found in this study. Most patients were immunocompetent hosts, suggesting that immune status is not a determining factor for susceptibility to sporotrichosis.

A history of direct contact with cats that had current sporotrichosis diagnoses was reported by 21 (42.8%) of the 49 patients, suggesting that zoonotic transmission from cats was likely for those particular cases. Cats are the principal transmission vector leading to sporotrichosis outbreaks in many countries, particularly in South America and some countries in Asia (*16*,*33*,*39*). Claws, skin lesions, nasal and oral cavities, and feces of infected cats contain a considerably higher number of fungi than environmental sources, suggesting that *Sporothrix* spp. transmission more likely occurs from infected cats than from environmental sources (*40*). The rising incidence of the disease in humans aligns with the previously reported outbreak of feline sporotrichosis in Thailand (*27*). Although insects are known to be carriers of *Sporothrix* spp. fungi (*41*), a history of insect bites in patients with sporotrichosis has rarely been reported. In this study, 3 patients had lesions located in the area of previous insect bites. Sapronosis is another commonly cited primary mode of transmission in sporotrichosis outbreak studies (*35*). However, only 1 patient in this study reported possible sapronotic transmission from contact with contaminated soil after a bicycle fall.

The upper extremities have been ranked first and lower extremities ranked second as the most common sites for *Sporothrix* lesions (*29*,*35*,*36*). Moreover, lesions in the upper extremities are more likely to be reported in patients exposed to cats. Most sporotrichosis manifestations were lymphocutaneous or fixed cutaneous forms and occurred in similar proportions, aligning with our results. Disseminated sporotrichosis has rarely been reported globally (*29*,*37*,*38*) and has not been reported in Thailand (*29*).

Patients >60 years of age tended to have nonpustular lesions more frequently than did younger persons. This age-related pattern could be caused by immunosenescence, which is characterized by alterations in innate and adaptive immunity (*42**,**43*). As a person ages, their immunologic functions gradually decline, contributing to poor responses and diminished levels of inflammation after new infections (*42*,*43*).

Different *Sporothrix* species are predominant in various regions worldwide. *S. schenckii* is the primary species causing sporotrichosis in Australia, America, and South Africa, whereas *S. globosa* is the etiologic agent in most patients in China (*4*,*24*). *S. brasiliensis* is the main organism causing feline and human sporotrichosis in Brazil (*4*,*44*). Feline sporotrichosis caused by *S. schenckii* s.s. has been reported in southern Thailand, which suggests species transmission to humans (*27*). In Thailand, further studies and molecular identification of species causing human sporotrichosis are needed to explain the epidemiology and association with feline sporotrichosis. This study only reported the *S. schenckii* complex by using fungal culture and colony morphologic identification during routine laboratory investigations in clinical practice. 

No evidence of fungal forms was seen in our cases during histopathologic examination; the absence of fungi in histopathologic sections remains unclear. However, on the basis of previous research, several factors might influence pathogen load in tissue biopsies. One factor is the onset or duration of the disease before the biopsy is obtained. A study of liver granuloma formation after *Histoplasma* sp. infection reported the highest fungal load in tissue at 7 days postinfection; substantial infection control was observed by 50 days (*45*). That finding suggests that early biopsy within the first week of symptoms might increase the likelihood of detecting the pathogen. Conversely, over time, granulomas might undergo structural changes that reduce their capacity to contain the infection (*46*). Another factor is the virulence of the pathogen; some fungi possess virulence factors that enable them to overcome host defenses (*47*). For example, *S. brasiliensis* has structural features that potentially inhibit and evade phagocytosis, contributing to its higher virulence than that for *S. schenckii* (*48*). Those factors might explain why our findings align with a study in Malaysia that reported low evidence of fungal organisms in tissues, rather than with higher detection rates observed in Brazil (*38*,*39*). Further studies involving larger populations are needed to explore those hypotheses.

Numerous treatments for cutaneous sporotrichosis are available. For example, oral antifungal agents, a saturated oral solution of potassium iodide, and excision can be used as standalone or combination therapies. However, oral itraconazole remains the treatment of choice, and the length of treatment is dependent upon lesion recovery (*4*,*24*,*36*). Despite the lack of a defined cutoff value for antifungal susceptibility among *Sporothrix* spp., our study found that all 9 isolates subjected to susceptibility testing exhibited an itraconazole MIC of <1 μg/mL. However, no significant difference was observed for itraconazole MICs between patients with and without treatment failure at a dose of 200 mg/day. Time-to-event analysis also showed no significant difference in time-to-improvement or time-to-cure between patients with a 1 μg/mL MIC and <1 μg/mL MIC, consistent with the study from Brazil (*25*). This study’s median duration until cure was 180 days, aligning with the findings from a 19-case review in Southeast Asia (*29*) and other studies (*49*,*50*). This study did not identify any clinical factors that influenced the duration until cure.

In conclusion, cutaneous sporotrichosis is a rare disease in Thailand. However, the number of cases has escalated dramatically since 2018, and ≈65% of patients have reported a history of cat exposure. Consequently, taking a thorough risk-factor history, especially regarding cat exposure, is crucial for guiding early clinical suspicion of disease and diagnosis. The standard for diagnosis is fungus isolation; the *S. schenckii* complex is suspected to be the primary sporotrichosis-causing species in Thailand. Itraconazole is the first-line treatment, and resistance to this drug has not been reported. Physicians and veterinary personnel should heighten their awareness of the sporotrichosis outbreak in Thailand to enable more effective disease control. In addition, early recognition of suspected sporotrichosis cases in cats, appropriate antifungal treatment, and education on how persons can avoid zoonotic transmission and manage owned or infected cats are all recommended to potentially minimize zoonotic transmission of sporotrichosis.
